# Palliative care in primary health care: *scoping review*

**DOI:** 10.1590/1518-8345.3858.3324

**Published:** 2020-07-01

**Authors:** Eveline Treméa Justino, Maristel Kasper, Karen da Silva Santos, Rita de Cassia Quaglio, Cinira Magali Fortuna

**Affiliations:** 1 Universidade Estadual do Oeste do Paraná, Curso de Enfermagem, Foz do Iguaçu, PR, Brazil.; 2 Scholarship holder at the Fundação Araucária, Foz do Iguaçu, PR, Brazil.; 3 Universidade de São Paulo, Escola de Enfermagem de Ribeirão Preto, PAHO/WHO Collaborating Centre at the Nursing Research Development, Ribeirão Preto, SP, Brazil.; 4 Scholarship holder at the Coordenação de Aperfeiçoamento de Pessoal de Nível Superior (CAPES), Brazil.; 5 Scholarship holder at the Fundação de Amparo à Pesquisa do Estado de São Paulo (FAPESP), Brazil.; 6 Universidade de São Paulo, Hospital da Clinicas da Faculdade de Medicina de Ribeirão Preto, Equipe Gestora de Neurologia e Equipe de Interconsulta de Cuidados Paliativos, Ribeirão Preto, SP, Brazil.

**Keywords:** Palliative Care, Terminal Care, Hospice Care, Critical Illness, Primary Health Care, Review, Cuidados Paliativos, Assistência Terminal, Cuidados Paliativos na Terminalidade da Vida, Estado Terminal, Atenção Primária à Saúde, Revisão, Cuidados Paliativos, Cuidado Terminal, Cuidados Paliativos al Final de la Vida, Enfermedad Crítica, Atención Primaria de Salud, Revisión

## Abstract

**Objective:**

to map the available evidence on the main topics investigated in palliative care in primary health care.

**Method:**

scoping review type study carried out in five databases, including original articles, based on the descriptors palliative care, palliative care at the end of life, terminal care, terminal state, primary health care and their respective acronyms and synonyms, totaling 18 publications. The extraction of data from primary studies was performed using an instrument produced by the authors and which allowed the construction of the categories presented.

**Results:**

18 publications were included in this review. Among the most studied themes are the difficulties of the teams regarding the continuity of care in the health network; the importance of in-service education by the multidisciplinary team; professional unpreparedness; bioethics; the validation and application of scales for prognosis and care for some pathologies such as cancer and diabetes; among others.

**Conclusion:**

it became evident that palliative care in primary health care has been gradually developed, but it is necessary to consider the organization of primary health care and the social policies that support or weaken it, being considered a complex challenge.

## Abstract (pt)

**Objetivo:**

mapear as evidências disponíveis sobre os principais temas investigados em cuidados paliativos na atenção primária à saúde.

**Método:**

estudo do tipo scoping review realizado em cinco bases de dados, sendo incluídos artigos originais, a partir dos descritores cuidados paliativos, cuidados paliativos na terminalidade da vida, assistência terminal, estado terminal, atenção primária à saúde e seus respectivos acrônimos e sinônimos, totalizando 18 publicações. A extração dos dados dos estudos primários foi realizada por meio de instrumento produzido pelas autoras e que permitiu construir as categorias apresentadas.

**Resultados:**

foram incluídas 18 publicações nesta revisão. Dentre os temas mais estudados estão as dificuldades das equipes quanto à continuidade de cuidados na rede de saúde; a importância da educação em serviço da equipe multiprofissional; o despreparo profissional; a bioética; a validação e aplicação de escalas para prognóstico e cuidados voltados para algumas patologias como câncer e diabetes; dentre outros.

**Conclusão:**

evidenciou-se que os cuidados paliativos na atenção primária à saúde vêm sendo gradativamente desenvolvidos, mas há que se considerar o modo de organização da atenção primária à saúde e as políticas sociais que as sustentam ou enfraquecem, sendo considerado um desafio complexo.

## Abstract (es)

**Objetivo:**

mapear las evidencias disponibles sobre los principales temas investigados sobre cuidados paliativos en la atención primaria a la salud.

**Método:**

estudio de tipo *scoping review*, realizado en cinco bases de datos, habiéndose incluido artículos originales, a partir de los descriptores: cuidados paliativos; cuidados paliativos en el término de la vida; asistencia terminal; estado terminal; y, atención primaria a la salud y sus respectivos acrónimos y sinónimos, totalizando 18 publicaciones. La extracción de los datos de los estudios primarios fue realizada por medio de instrumento producido por las autoras, él que permitió construir las categorías presentadas.

**Resultados:**

en esta revisión fueron incluidas 18 publicaciones. Entre los temas más estudiados están: dificultades de los equipos en cuanto a la continuidad de cuidados en la red de salud; importancia de la educación durante el servicio del equipo multiprofesional; falta de preparación profesional; bioética; validación y aplicación de escalas para pronóstico; y, cuidados dirigidos para algunas patologías como cáncer y diabetes - entre otros.

**Conclusión:**

se evidenció que los cuidados paliativos en la atención primaria a la salud están siendo gradualmente desarrollados, pero es necesario considerar el modo de organización de la atención primaria a la salud y las políticas sociales que las sustentan o debilitan; lo que es considerado un desafío complejo.

## Introduction

The most recent definition of Palliative Care (PC) was published in 2018 and developed after a large project involving more than 400 members from 88 countries of the International Association for Hospice & Palliative Care (IAHPC), an association that maintains close ties and official relations with the World Health Organization (WHO)^(^ 1 ^)^. Currently, PCs are defined as “active holistic care, offered to people of all ages who are in intense suffering related to their health, resulting from serious illness, especially those who are at the end of life. The objective of Palliative Care is, therefore, to improve the quality of life of patients, their families and their caregivers”^(^ 1 ^)^.

Thus, offering palliative care to people with serious illnesses takes into account not only the person, but everyone involved in the care. As a definition, serious illness is understood as “any acute or chronic illness and/or condition that causes a significant disability and that can lead to a condition of disability and/or weakness for a long period, or even death”^(^ 2 ^)^.

In Brazil, isolated discussions and initiatives in PC have been found since the 1970s. It is noteworthy, however, that it was in the 1990s that the first organized services began to appear^(^ 3 ^)^. It is noteworthy that, in the 1980s, the life expectancy of Brazilians was 62.5 years^(^ 4 ^)^. Currently, the reality is different, the numbers have increased, both life expectancy has risen to 76 years^(^ 4 ^)^ and the number of services that provide palliative care in the country. This increase in life expectancy is the result of investments in public policies by the State, including the implantation and implementation of the Unified Health System (UHS).

Until August 2018, after a survey carried out by the National Academy of Palliative Care (NAPC), 177 palliative care services were identified, distributed in the five regions of Brazil. Of these, 58% (103 services) are concentrated in the Southeast region, 20% (36 services), in the Northeast region, 14% (25 services), in the South region, 5% (eight services), in the Midwest region, while only 3% (five services) are located in the North of the country^(^ 5 ^)^.

As a result of the growth in the life expectancy of the population, there is an increase in the occurrence of Chronic-degenerative Noncommunicable Diseases (CNCDs), which make the demand for PC a contemporary public health problem. An integrative review^(^ 6 ^)^ on primary health care (PHC) and PC, held in 2014, aimed to understand the roles of PHC professionals in palliative care and pointed out that PHC can make a difference to patients and their families by having easy access to them, close to home, be able to carry out constant management of symptoms and sensitivity to the realities of the community: “Professionals monitor the aging and fragility of their patients, the efforts of families to accommodate the new care needs of their members, fears, financial insecurity and, therefore, cannot avoid this moment: it is exactly in situations like this that the full potential of PHC actions and the Family Health Strategy (FHS) becomes more evident. No other health service can stand side by side with these families with such property and face the path of palliation with constant presence, guidance and welcome”^(^ 6 ^)^.

A systematic review^(^ 7 ^)^, held in 2015 on palliative care and PHC, aimed to identify, in the view of health professionals, the ethical problems arising from practice in this context. The ethical problems detected were the scarcity of resources, the lack of knowledge about PC, the lack of communication skills, the difficulty of establishing limits in the clinical relationship, the work overload and the lack of support from the reference services. The authors concluded that, in order to incorporate PCs in PHC, specific norms and training are required, in addition to the culture of shared and co-responsible care^(^ 7 ^)^.

Therefore, in order to know the current panorama of palliative care in PHC and because there are no studies like this, which synthesize scientific evidence in relation to the theme, justifying this study and its importance, it is presented as an objective: to map the available evidence on the main topics investigated in palliative care in primary health care.

## Method

This is a scoping review study, carried out based on a set of techniques in order to map knowledge on certain topics in a research field. It differs from systematic review because it aims to focus on broader themes and include studies with different designs^(^ 8 ^)^. The elaboration of the scoping review followed a process consisting of five stages: a) Identifying the research questions; b) Identify the relevant studies valid for the investigation; c) Selection of review studies; d) Mapping of data from studies included in the review; e) Confront, summarize and report the results^(^ 8 ^)^.

For this, the PICO strategy was used, acronym for Patient, Intervention, Comparison and Outcomes, for the definition of the following guiding question: “What evidence is available about palliative care in primary health care?”. For the construction of the aforementioned question and to carry out the search, the PICO strategy was stipulated as: P for Population, Patient or Problem (what evidence is available); I of Interest (palliative care) and Context CO (primary health care).

This review was conducted in the databases Medical Literature Analyzes and Retrieval System Online (PubMed/MEDLINE), Latin American and Caribbean Literature in Health Sciences (LILACS), Web of Science, Scopus and Cumulative Index to Nursing & Allied Health Literature (CINAHL). For this, the controlled and indexed descriptors were used for each of the databases in this review. For the combination of these, the boolean operators OR and AND were used.

From the research question, terms were selected in the Health Sciences Descriptors (DeCS) and terms in the Medical Subject Headings (MeSH) containing the appropriate descriptors for searching the databases. The controlled descriptors used were as follows: a) PubMed/MEDLINE: palliative care (synonym: palliative treatment), hospice care (synonym: hospice program), terminal care, critical illness, primary health care (MeSH); b) LILACS: palliative care (synonyms: palliative care, palliative treatment), palliative care at the end of life (synonyms: terminally ill care, palliative care for terminally ill patients, comfort care, intermittent care program, palliative care program), terminal assistance (synonym: terminal care), terminal care (synonym: terminal illness), Primary Health Care (synonyms: primary care, primary care in health, primary health care, basic care, basic health care, basic care in health, basic care, primary care, primary health care, primary care, primary health care and primary health care (DeCS); c) Web of Science; d) Scopus; e) CINAHL: Palliative care (synonym: palliative treatment), hospice care (synonym: hospice program), terminal care, critical illness, primary health care (MeSH).

For the selection of studies, the following inclusion criteria were considered: a) original research articles; b) made available in the full version; c) published in the period 2009 to 2019, the last ten years; d) Portuguese, French, Spanish and English languages.

Exclusion criteria were defined: a) studies whose theme palliative care is presented as a “recommendation” in the results and conclusions, not being configured as a central object of the study; b) dissertations, theses, public policies and videos. The search for jobs for primary research took place on April 27, 2019 via advanced form.

After identification, primary studies were selected, according to the guiding question and the previously defined inclusion and exclusion criteria. This step was carried out by two reviewers independently. The instrument, designed for the purpose of extracting and analyzing data from the included studies, was composed of the following items: 1- article identification; 2- object and/or question and/or objectives of the study; 3- type of study/design; 4- data production tools and/or techniques; 5- year; 6- magazine; 7- participants and/or sample; 8- main results; 9- area; 10- country. The stages of selection of the studies included the identification, screening, eligibility and inclusion.

The categorization was the form adopted for the analysis of the results in which it was extracted how the PC has been carried out in the PHC. The work of analyzing the studies consisted of a careful task. There was an interest in knowing and characterizing the panorama of the studies according to its investigated object. For the presentation of the data, we chose to build sequential figures that sought to demonstrate how the CP theme in PHC was studied by different authors.

## Results

**Figure 1:**
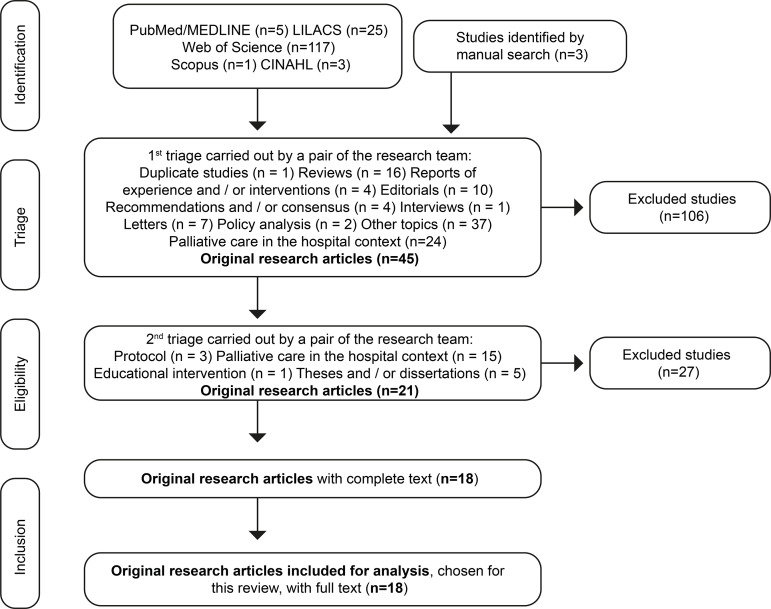
Flowchart of the study selection process for the review of the adapted scope of the Preferred Reporting Items for Systematic Review and Meta-Analyses (PRISMA). Ribeirão Preto, SP, Brazil, 2019

In the research, a total of 154 studies were found, 25 in LILACS, 112 in Web of Science, five in PubMed/MEDLINE, three in CINAHL, one in Scopus and three were identified by manual search. After proceeding with the inclusion and exclusion criteria, two successive evaluations and disregarding duplicate articles, 18 publications^(^ 9 ^-^ 26 ^)^ were relevant for this review, since they met the study question and pre-established criteria, as explained in the analysis flowchart (Figure 1).

With regard to language, eleven studies analyzed were published in Portuguese^(^ 9 ^-^ 11 ^,^ 18 ^-^ 21 ^,^ 23 ^-^ 26 ^)^ and from Brazilian authors, five were published in Spanish^(^ 12 ^-^ 13 ^,^ 15 ^-^ 17 ^)^ and three in English^(^ 14 ^,^ 16 ^,^ 22 ^)^. Of the included studies, eight^(^ 9 ^-^ 11 ^,^ 17 ^-^ 21 ^)^ used the qualitative approach and ten, the quantitative^(^ 12 ^-^ 16 ^,^ 22 ^-^ 26 ^)^. As for the instruments / techniques for data collection/production, questionnaires, interviews, observation, discussion groups and application of scales were used. Among the study participants, the following were identified: health professionals; formal and informal caregivers; patients eligible for palliative care. Two studies, also, had sample of medical records of patients, according to Figure 2.

**Figure 2:** Summary of articles with authors, magazines and countries, type of study, instruments and/or techniques for data production, participants and/or sample of articles analyzed. Ribeirão Preto, SP, Brazil, 2019

Article	Journal/Country	Study design	Participants and/orsample
Being cared for by a relative: existential feelings of cancer patients^(^ 9 ^)^	Texto & Contexto - Enfermagem / Brazil	- Qualitative - Interviews	20 patients
Meaning of being a caregiver of a family member with cancer and dependents: contributions to palliation^(^ 10 ^)^		- Qualitative - Interviews	17 family caregivers
Meanings attributed by health professionals to palliative care in the context of primary care^(^ 11 ^)^		- Qualitative - Interviews	25 professionals from health units and the Family Health Support Center (FHSC*)
Diagnosis of tumor asthenia in Primary Care. Proposal of correlation between of the scales^(^ 12 ^)^	Medicina Paliativa / Spain	- Observational, descriptive study - Computerized clinical history (DIRAYA^†^) during consultations and home visits	67 patients
Validation of a prognostic model of survival based on biological parameters for terminal cancer patients cared for at home^(^ 13 ^)^		- Quantitative - Analytical, observational and prospective study - Clinical history from home visit	80 patients
Identification and characteristics of patients with palliative care needs in Brazilian primary care^(^ 14 ^)^	BMC Palliative Care/Australia	- Quantitative - Cross-sectional study - Questionaire	238 patients
Prevalence of pain as a reason for consultation and its influence on sleep: experience in a primary care center^(^ 15 ^)^	Atención Primaria/ Spain	- Cross-sectional description - Questionaire - Pain scale application - Clinical interview	206 patients
A new measure of home care patients' dignity at the end of life: The Palliative Patients' Dignity Scale (PPDS^¶^)^(^ 16 ^)^	Palliative and Supportive Care/ Spain	- Quantitative - Questionaire	80 participants, including patients, family caregivers and professionals
Sick people at the end of life: experiences in the accessibility to social health resources^(^ 17 ^)^	Enfermería Universitaria / Spain	- Qualitative - Discussion grups - Interviews	41 caregivers
(In defense of) Palliative Care in Primary Health Care^(^ 18 ^)^	O Mundo da Saúde / Brazil	- Qualitative - Interviews	11 health professionals linked to the Family Health Strategy (FHS^‡^)
Perception of family members and health professionals about end-of-life care in the context of primary health care^(^ 19 ^)^	Ciência & Saúde Coletiva / Brazil	- Qualitative - Interviews	Seven family members; three FHS professionals^‡^; two professionals from the Home Care Program
Palliative care in home care: the perspective of occupational therapists^(^ 20 ^)^	Cadernos de Terapia Ocupacional / Brazil	- Qualitative - Interviews	Eight occupational therapists
Palliative care in primary health care: ethical considerations^(^ 21 ^)^	Revista Bioética / Brazil	- Qualitative - Case study - Interviews - Analysis of medical records - Home visit	Seven FHS teams ^‡^ Two patients in palliative care
New demands for primary health care in Brazil: palliative care^(^ 22 ^)^	Investigación y educación en enfermería / Colombia	- Quantitative - Descriptive - Application of the Karnofsky Performance Scale (KPS^§^)	160 medical records
Comfort of formal and informal caregivers of patients in palliative care in primary health care^(^ 23 ^)^	Revista Rene/ Brazil	- Quantitative - Cross-sectional study - Questionaire	50 caregivers of patients in palliative care
Perspectives for palliative care in primary health care: a descriptive study^(^ 24 ^)^	Online Brazil Journal of Nursing/Brazil	- Documental - Descriptive - Application of KPS^§^	2715 medical records
Interface between social support, quality of life and depression in users eligible for palliative care^(^ 25 ^)^	Revista da Escola de Enfermagem da USP /Brazil	- Quantitative - Cross-sectional Correlational - Interviews - Application of KPS^§^	687 patients
Identifying patients for palliative care in primary care in Brazil: experience of the Being at Your Side Project^(^ 26 ^)^	Revista Brasileira de Medicina de Familia e Comunidade / Brazil	- Quantitative - Application of Supportive and Palliative Care Indicators Tool (SPICT**)	38 patients

* FHSC = Family Health Support Center;

† DIRAYA = Computerized clinical history;

‡ FHT = Family Health Teams;

§ KPS = Karnofsky Performance Scale;

¶ PPDS = Palliative Patients' Dignity Scale;

** SPICT = Supportive and Palliative Care Indicators Tool.

The context of the studies was variable. Several experiences were found, such as: in daily life and patient care at home^(^ 9 ^,^ 19 ^)^; meaning of being a caregiver and on PC^(^ 10 ^,^ 11 ^)^ for health professionals; evaluation of tumor asthenia^(^ 12 ^)^; validation and application of a tool for prognosis^(^ 13 ^)^; identification and characterization of patients in PC and services^(^ 14 ^,^ 22 ^,^ 24 ^,^ 26 ^)^; pain evaluation^(^ 15 ^)^; assessment of dignity at the end of life^(^ 16 ^)^; difficulties in accessing PCs^(^ 17 ^)^; the work process in PHC^(^ 18 ^)^; perspectives of occupational therapists in PC in PHC^(^ 20 ^)^; ethical problems^(^ 21 ^)^; comfort of caregivers of patients in PC^(^ 23 ^)^; relationship between social support, quality of life and depression in PC patients in PHC^(^ 25 ^)^(Figure 3).

**Figure 3:** Synthesis with object and/or question and/or objectives of the studies and main results found in the analyzed articles. Ribeirão Preto, SP, Brazil, 2019

Objectives	Main results
Understand the daily life of cancer patients in palliative care when experiencing the care of their family at home^(^ 9 ^)^.	Patients who received authentic care from their families reflect the impact they had, even in the face of the mishaps that have grown and transcended. Home care, combined with palliative care, is capable of giving "wings" to those who viewed their lives as threatened.
Understand the meaning of being a caregiver for a family member with cancer and with high dependence for daily activities^(^ 10 ^)^.	It meant, for the caregiver, to be terrified with the diagnosis, with the treatment, with palliative care and being-with-the-other in the disease. He showed himself to be busy while remaining concerned and helpful. Palliative care must permeate the nurse's work so that it is a true being-of-care.
Understand the meanings attributed by health professionals to palliative care assistance in Primary Health Care^(^ 11 ^)^.	The professionals recognized the need for the other in palliative care in primary care. The meanings involved the need for a system organized in a network that favors social relations, coping with the curative hospital-centered model and the inclusion and awareness of the family.
Detect tumor asthenia in Primary Care, evaluation possibilities and its gradation with two proposed scales, the adapted ICD*-10 (ICD*-10) classification and the graduated Karnosfky index (IK^†^)^(^ 12 ^)^.	The study confirmed the existence of a negative correlation or inverse relationship between the degrees of tumor asthenia, according to the diagnostic criteria of ICD*-10, for tumor asthenia against IK^†^. Easy and accessible tool in any care environment, including primary and palliative care, not only a functional indicator, but can also assess and grade tumor asthenia.
To verify the validity and application of a pronounced tool developed in the hospital with biological parameters for its application at home^(^ 13 ^)^.	At home, the routine use of biological parameters of peripheral blood for prognostic purposes is of little use. The use of easily registered variables (clinical symptoms, functional status and aspects related to treatment) can be a more adequate tool to estimate survival in this environment.
Identify how many patients in the Brazilian FHS^‡^ program have needs for PC^§^; Describe the health conditions and sociodemographic status of patients in the FHS^‡^ program with PC^§^ needs; Describe the professional and social support received by patients in the FHS^‡^ program with PC^§^ needs^(^ 14 ^)^.	Patients with PC^§^ needs are accessing the FHS‡ program, regardless of whether there is specific support for PC^§^. Of the 238 identified patients, 73 were identified as needing PC^§^, and the average age was 77.18. Most patients received medication and professional support through primary care units, but limitations of services were identified, such as lack of home visits and limited multiprofessional approaches. PC^§^ policies and professional training must be implemented to improve this area.
Determine the frequency of pain as a reason for visiting in a primary care consultation and knowing your influence^(^ 15 ^)^.	Average age of 50 years, 56% women. Pain intensity with the VAS^||^ scale was 4.9. 45% of patients who met the criteria for "good sleep". In men, acute pain and its intensity appeared as independent factors of bad sleep.
Develop a new and brief instrument to be employed in dignity measurement, one based on the perceptions of patients, relatives, and professionals about dignity^(^ 16 ^)^.	An eight-item questionnaire was presented for validation. The new scale showed appropriate factor validity and reliability. The new PPDS^¶^ has appropriate psychometric properties that, together with its brevity, encourage its applicability in assessing dignity at the end of life.
Identify the main limitations and difficulties in accessing socio-health resources that people have lived at the end of life, through the experiences and perceptions of the caregivers of these patients^(^ 17 ^)^.	Categories: food, emergency services, need for privacy, feeling of solidity and experience at home. Obstacles: care protocols that do not arise in the family process or adoration process and the need for an individualized room in the hospital. At home, they are protected by the profession of basic care, but they present difficulties, not access to psychological support and palliative care units.
Analyze the work process of health professionals who work in Family Health / Primary Care and have already taken care of people in the process of dying in order to outline possible contributions to the area of Public Health with regard to the implementation of palliative care in Attention Primary^(^ 18 ^)^.	The planning of health actions is guided by the Singular Therapeutic Project, with an emphasis on social diagnosis and the need for a bond to agree. It is understood that the purpose of this activity of the health professional is to promote dignity and quality of life in the death process, however, it is argued that comprehensive care must include, in addition to the care of the person and his family, the defense of full human development.
Reflect on the care of people with terminal illnesses in Primary Health Care (PHC**)^(^ 19 ^)^.	Categories: care at the end of life in the perception of family members and health professionals. Despite the humanization discourse, care was discontinued. Apart from large centers, there is little improvement in the quality of life of those who die at home.
To know and reflect on the perspectives of occupational therapists in relation to implementation of palliative care in home care^(^ 20 ^)^.	Professional performance in palliative care concentrated at specialized levels, but with power in primary and home care. Barriers: complexity of "being at home", high cost demands, lack of infrastructure and the failure to implement public policies. Insufficient professional training and scientific production.
Identify cases of users in order to inventory the ethical problems that the team experiences^(^ 21 ^)^.	The training of human resources with technical competence and the continuity of assistance in the transition from curative to palliative treatment favors comprehensiveness and obtaining more appropriate responses to ethical challenges. It is concerned with the identification of the values underlying the specific needs of the end of life and with a multidisciplinary approach.
Assess the need for incorporation of palliative care in primary health care through the characterization of users eligible for this type of care, enrolled in a program for devices dispensing^(^ 22 ^)^.	141 of the 160 selected medical records had KPS^††^ information. Most cases performed below 70% and, therefore, patients were eligible for palliative care. The most frequent pathologies are chronic degenerative diseases.
Analyze the comfort of formal and informal caregivers of patients in palliative care, identifying the variables associated with difficulties in home care^(^ 23 ^)^.	Most caregivers were women, average age 52 years old, with companions and practitioners of some religion. The comfort level of caregivers of patients in palliative care was relatively good and was associated with difficulties in home care.
Identify patients eligible for palliative care and characterize the services involved in Primary Health Care^(^ 24 ^)^.	It is reported that 2715 are eligible, representing 3.59% of the registered population; cardiovascular diseases, diabetes and cancer; 17.2% required early palliative care; 9.7%, exclusive. Need to structure PHC** for early palliative care, focusing on the elderly.
To analyze the relationship between social support, quality of life and depression in patients eligible for palliative care seen at PHC** in a municipality in the interior of Minas Gerais, Brazil^(^ 25 ^)^.	Higher levels of social support are related to patients with better overall and functional quality of life. On the other hand, lower levels of quality of life due to the presence of physical symptoms are related to worse levels of social support, and a worse overall quality of life is related to higher levels of symptoms of depression.
To present the process of identification of palliative care patients in a Family Health Strategy´s team in Brazil^(^ 26 ^)^.	38 people with palliative needs were identified out of a population of 3,000; 58% are women; 63% are over 65 years old. There is greater multimorbidity over 65 years. Cardiovascular, respiratory, psychiatric, cancer and Diabetes Mellitus are prevalent.

* ICD = International Classification of Diseases;

† KI = Karnofsky Index;

‡ FHS = Family Health Teams;

§ PC = Palliative Care;

|| VAS = Visual Analysis Scale;

¶ PPDS = Palliative Patients' Dignity Scale;

** PHC = Primary Health Care;

†† KPS = Karnofsky Performance Scale

## Discussion

The analysis of scientific production has shown that, although there have been advances in recent years, palliative care practices in PHC have still been incipient and, when they occur, present limits, as described in the results in figure 3, as discontinuity of care^(^ 13 ^,^ 21 ^)^, the complexity and/or difficulties in palliative care at home^(^ 19 ^-^ 20 ^,^ 23 ^)^, the peculiarities of palliative care with high cost demands - infrastructure^(^ 20 ^)^, the insufficient number of visits by health professionals^(^ 14 ^)^, limited multidisciplinary approaches^(^ 14 ^)^, insufficient professional training^(^ 14 ^,^ 20 ^-^ 21 ^)^, the reduced scientific production in the area^(^ 21 ^)^, the need to structure PHC for this purpose^(^ 24 ^)^,the existence of very general care protocols^(^ 17 ^)^ and difficulties in accessing psychological support^(^ 17 ^)^.

Such aspects are related to the organization of care networks and primary health care. In Brazil, Family Health teams represented advances in access and health indicators, but face difficulties of various kinds, such as economics, with underfunding of health, training of professionals and improvement of network articulation, among others^(^ 27 ^)^.

An analysis of the current panorama of the PC, carried out in 2018, indicated that, although aware that primary care may be the strategy with the lowest cost and greatest impact on the health of a population, the provision of palliative care in the country is centered on hospitals^(^ 5 ^)^. Perhaps, the incipience of this practice in Brazilian services is demonstrated by the absence of a public health policy that specifically structures or guides the development of these actions^(^ 5 ^)^.

A study involving Brazil and France addressed the transition of patients between hospital palliative care services and homes and showed that, in both countries, there are difficulties in this transition related, among others, to caregiver fatigue and fear of death. In this study, hospital discharge was also addressed with rationalizing purposes such as the release of beds^(^ 28 ^)^.

The studies^(^ 14 ^,^ 20 ^-^ 21 ^)^ addressed the insufficient training of professionals for palliative care and this theme was also evidenced in research^(^ 7 ^)^, pointing out that the professionals’ unpreparedness to deal with the demands and needs of chronic health conditions was present in countries that have an aging population longer than Brazil.

Death, understood as a theme that is part of the daily life of health services, integrates the different phases of the human life cycle, but it is still a topic that is treated in a reduced way, both in the training of health professionals and in health services, compromising the principle of integrality.

In the study^(^ 11 ^)^ it was shown that the request by family members not to reveal the diagnosis to the patient may coincide with the professional’s option, reinforcing their own difficulties with the subject. An integrative review of PC in PHC highlighted that the monitoring of the process of death and grief and the communication of bad news, among other topics, are rarely addressed in training and health services^(^ 7 ^)^.

In some speeches of the professionals and in their actions, the curative model still prevails, focused on the disease and the specificity of care. The training of professionals could favor health education for patients and families, especially in relation to the implementation of PC in PHC^(^ 11 ^,^ 14 ^)^.

In addition, Permanent Health Education (PHE) is considered learning at work, takes everyday life as an open space and a permanent reviewer of professional practices, as a place of subjectivity and discussions^(^ 29 ^)^. Thus, it is believed that institutionalized spaces that can put professional practices in an interdisciplinary way, are essential for the promotion of comprehensive palliative care.

However, it is considered that the provision of palliative care in primary care is related not only to the professionals’ capacity for the development of palliative care, but also with universal health systems that are organized in a network and guided by an expanded conception of the health-disease process, taking into account social determinants and social inequalities. “Poor” teams in poor locations that materialize selective primary care are unable to take on the complexity of primary health care and palliative care.

Teams without adequate working conditions may, for example, make an insufficient number of visits by health professionals to these families^(^ 14 ^)^.

A study^(^ 30 ^)^ on Primary Health Care in Latin America addressed 12 countries and discussed the relationship between the implementation of comprehensive primary care and universal social protection. Certainly, this aspect does not match the implementation of neoliberal policies and flexibility of social rights, as has been seen not only in Brazil.

The authors addressed Primary Health Care in full, which considers the family and community focus, the territorial base, the work in multi-professional teams and social participation and the expansion of this aspect in Latin America in left and center-left governments. Concerns were raised about the current political scenario in this region of the world.

It was observed that five^(^ 9 ^-^ 10 ^,^ 12 ^-^ 13 ^,^ 23 ^)^ articles brought the association of palliative care and cancer. Another five^(^ 16 ^,^ 22 ^,^ 24 ^-^ 26 ^)^ articles mentioned cancer, but not in isolation, they also pointed out other diseases such as diabetes, Alzheimer’s disease, as well as respiratory and cardiovascular diseases. The expansion of PCs to other diseases appeared in a study on human rabies and suggested a review of clinical guidelines, proposing the introduction of PCs for people with rabies in endemic countries^(^ 31 ^)^.

Initially, palliative practice was directed only to cancer patients, but gradually it started to be incorporated by other specialties involved in the care of patients with chronic-degenerative diseases. In its first edition, published in 1995, the “Medical guidelines of the National Palliative Care Organization (NPCO) for determining the prognosis in Selected Non-Cancerous Diseases” determined the prognosis of non-cancer diseases and included them in the palliative care programs. The first non-cancer diseases to be included were: Congestive Heart Failure (CHF), Chronic Obstructive Pulmonary Disease (COPD) and Alzheimer’s disease^(^ 32 ^)^.

Subsequently, the second edition, published in 1996, added the following pathologies: Aids (Acquired Immunodeficiency Syndrome); liver and kidney disease; leakage; coma and Amyotrophic Lateral Sclerosis (ALS)^(^ 32 ^)^.

If the association of diseases with palliative care can facilitate the specificity of the care to be offered, for Primary Health Care teams, other knowledge needs to be mobilized, such as, for example, the dynamics of family relationships and the production of unique therapeutic projects, as pointed out in a study^(^ 18 ^)^.

The use of scales/instruments to define people eligible for palliative care was addressed in six articles, using the Palliative Performance Scale (PPS)^(^ 10 ^)^ and the Karnofsky Performance Scale (KPS)^(^ 14 ^,^ 22 ^,^ 24 ^-^ 26 ^)^. Another article proposed the correlation between two scales, the KPS and the ICD-10 classification (ICD-10)^(^ 12 ^)^.

In addition to the scales to identify people’s eligibility for palliative care, other instruments, such as the Visual Analog Scale (VAS), were used to assess pain intensity^(^ 15 ^)^, the General Comfort Questionnaire (GCQ), to assess the comfort of formal and informal caregivers^(^ 23 ^)^, the European Cancer Research and Treatment Organization scale (EORTC QLQ-C15-PAL), to measure patients’ quality of life, the Medical Outcome Study (MOS) scale, for assessing the level of social support, and the Center for Epidemiological Studies - Depression (CES-D) scale for identifying symptoms of depression^(^ 25 ^)^.

An article that aimed to develop the Dignity Scale for Palliative Patients (PPDS) and used six other scales is highlighted: the Patient Dignity Inventory (PDI), which identifies sources of suffering in patients at the end of life; the Hospital Anxiety and Depression Scale (HADS); the Brief Resilient Coping Scale (BRCS), which measures resilience; the GES Questionnaire, referring to spirituality; the IBPC C-30 Quality of Life scale (EORTC-QLQ-C30) and the Duke-UNC-11 Functional Social Support Questionnaire, which assesses confidential and affective social support^(^ 16 ^)^.

In addition, it is essential that the primary health care professional knows and knows how to use the main scales used in palliative care, such as the Edmonton Symptom Assessment Scale, the Karnofsky Performance Scale and the Palliative Performance Scale. Thus, professionals can have tools to help guide the specific care plan for palliative care.

Regarding the work process of FHS professionals involved in the care of patients at the end of the process of living, the following ways of caring can be identified: compassion-empathy^(^ 9 ^-^ 10 ^,^ 18 ^-^ 19 ^)^; respect and willingness to understand the meaning attributed by the patient and their family about death^(^ 10 ^,^ 17 ^,^ 21 ^)^; communication used as a strategy to establish a bond^(^ 9 ^-^ 10 ^,^ 18 ^-^ 19 ^,^ 21 ^,^ 24 ^)^; active and sensitive listening^(^ 9 ^-^ 10 ^,^ 18 ^,^ 21 ^)^; non-judgment or abandonment of the patient and maintenance of hope^(^ 10 ^,^ 19 ^)^, even about the efficiency of the treatment for symptom relief. This demonstrates that the way of doing, at this moment, emphasizes the ethical and interpersonal dimensions in the professional-patient-family relationship^(^ 18 ^)^.

The discussion on bioethics was not a recurring theme in the selected articles and, in only two^(^ 18 ^,^ 21 ^)^ publications, this subject was addressed. In one of^(^ 21 ^)^ them, ethics in palliative care was the central theme, including aspects involving the communication of bad news, such as lack of sincerity and hiding the truth. Another article^(^ 18 ^)^ approached the subject, presenting a brief discussion about otherness, considered one of the references of bioethics.

It should be added that, for the effectiveness and guarantee of palliative care in primary care, it is assumed that there is articulation between the various health services and several other sectors - consequently, other professional categories - such as: transport, to ensure accessibility; social security, guaranteeing social rights; and justice, to ensure access and equity.

In addition, in the intra-sectoral prism of the health system, relations with the various medical specialties (Geriatrics, Neurology, Psychiatry, Cardiology) must be signed and perpetuated, in addition to different multi-professional categories, areas of epidemiological information and management, among others^(^ 33 ^)^.

The importance of the multi-professional team was mentioned as fundamental in five^(^ 11 ^,^ 14 ^,^ 19 ^-^ 21 ^)^ articles, with an emphasis on the need for theoretical, scientific knowledge and specific clinical skills in the domain of different professions so that the integrality of actions in the process of offering palliative care takes place, thus contemplating the physical, psychosocial and spiritual dimensions of the patient and their family.

In addition, within its scope, PHC has the potential to develop a set of interventions that favor the quality of life and continuity of palliative care, inside and outside the home, and can potentially favor and provide the patient with care close to the patient family and friends, in addition to reducing the risk of infections and suffering from unnecessary hospitalizations^(^ 11 ^,^ 34 ^)^.

In addition, for the PC to evolve in PHC, it is necessary to plan the offer, identify and meet the needs, the available resources, practice the sharing of information through appropriate communication and define the commitments of the parties involved. This is done in collaborative relationships and practices between different professionals, families and managers.

In line with Resolution No. 41, of October 31, 2018, which provides for guidelines for the organization of palliative care, in the light of integrated continuous care, within the scope of the Unified Health System (UHS), it is advised that “the palliative care should be part of the integrated continuous care offered within the scope of the Health Care Network (HCN)”, with PHC being considered the originator of care and territorial action^(^ 33 ^)^.

In order for changes to occur in the scope of management and care, the ability to dialogue and problematize the current concepts within each health team is essential. The construction of new pacts, with the approximation of concepts about comprehensive, humanized and quality care, in addition to equity and milestones that occurred strongly in the process of reforming the Brazilian health system, are essential for such changes to occur^(^ 29 ^)^.

This study has as its limit the non-problematization of the different health systems where the studies were produced, and primary health care is not always developed in its integral aspect, and there is still a strong orientation of health services according to the hospital logic. In addition, another limitation is the inclusion of articles in only four languages (Portuguese, French, Spanish and English), which may have limited access to other publications on the topic.

It is hoped that the compilation of the findings presented in this review may give rise to new lines of research and encourage other publications, contributing to the scientific advancement of the theme and performing the function of aid and support for the restructuring of practices and policies related to palliative care in primary health care.

## Conclusion

The evidence and themes investigated relating palliative care in primary health care point to the possibility of this care, since these teams work closely with families and the territories where they live. Such a process could occur in health systems that implement primary care articulated with social policies, which ensure health as a human right, being difficult to implement in non-universal systems and with primary care teams with few resources and poor articulation in a service network.

The studies point out possibilities of using scales that can assist in the identification and follow-up of people in palliative care, such as the Edmonton Symptom Assessment Scale, the Karnofsky Performance Scale and the Palliative Performance Scale. There are also productions that point out aspects of subjectivity as important, highlighting empathy, listening and valuing cultural aspects.

There are articles that deal with specific pathologies, with emphasis on cancer, diabetes and several pathologies that participate in the epidemiological and demographic transition. It is emphasized that specific care needs to be recognized at the same time that, in primary health care, other knowledge needs to be mobilized to consider family and social dynamics and thus build unique therapeutic projects.

It was possible to identify, in this review, that there is a concern with the initial training of health professionals and on-the-job training, as this is still incipient. To improve this situation, the insertion of the PC discipline in health courses is recommended and the implementation of permanent education and health education actions to bring family members and professionals closer together, taking into account cultural and social aspects of each family and team.

However, it is reaffirmed that the provision of palliative care in primary care is a complex challenge that goes beyond the preparation of professionals and family members, involves changing the logic and the care model that is still centered on diseases, economic logic and professional practices that compete with each other in a corporate way. This process is also related to social policies that conflict with the implementation of neoliberal policies and the flexibilization of social rights.
